# Beyond Pairwise Interactions: The Totally Antisymmetric Part of the Bispectrum as Coupling Measure of at Least Three Interacting Sources

**DOI:** 10.3389/fninf.2020.573750

**Published:** 2020-10-26

**Authors:** Sarah Bartz, Christina Andreou, Guido Nolte

**Affiliations:** ^1^Department of Neurophysiology and Pathophysiology, University Medical Center Hamburg-Eppendorf, Hamburg, Germany; ^2^Machine Learning Laboratory, Berlin Institute of Technology, Berlin, Germany; ^3^Translational Psychiatry Unit, Department of Psychiatry and Psychotherapy, University of Lübeck, Lübeck, Germany

**Keywords:** bicoherence, EEG, MEG, artifacts of volume conduction, cross-frequency coupling

## Abstract

In this paper we make two contributions to the analysis of brain oscillations with CFC techniques. First, we introduce a new bispectral CFC measure which is selective to couplings between three or more brain sources. This measure can be derived from ordinary cross-bispectra by performing a total-antisymmetrization operation on them. Significant coupling values can then be attributed to at least three interacting signals. This selectivity to the number of sources can be helpful to test hypotheses on the number of brain sources involved in the generation of commonly observed brain oscillations, such as the alpha rhythm. In a second step we present the correct empirical distribution for the coupling measure, which is necessary to properly assess the significance of coupling results. More importantly however, this corrected statistic is not limited to our particular measure, but holds for all complex-valued coupling estimators. We illustrate how the very common misassumption of empirical normality of such estimators can lead to a systematic underestimation of *p*-values, the breakdown of multiple comparison control procedures and in consequence a drastic inflation of the number of false positives.

## 1. Introduction

Neural oscillations continue to be widely studied for their role in cognition and as a correlate of various neurophysiological states (Engel et al., 2013). Such neurophysiological processes exhibit high temporal dynamics that can best be captured by techniques providing the required temporal resolution. Even though EEG and MEG meet these requirements, volume conduction effects complicate the interpretation of sensor-level results in terms of the underlying brain sources.

Many connectivity measures attempt to distinguish true from artifactual connectivity by assuming a linear and approximately instantaneous mapping from sources to sensors (Nolte et al., 2004; Pascual-Marqui, 2007; Stam et al., 2007; Pascual-Marqui et al., 2011; Vinck et al., 2011; Hipp et al., 2012; Brookes et al., 2014; Chella et al., 2014). While most of the above references detect linear coupling at one specific frequency, measures for cross-frequency coupling (CFC) exist in the form of the cross-bispectrum or its normalized version (bicoherence), which are third-order statistical moments in the frequency domain (Chella et al., 2014; Bartz et al., 2019). In its most general form, the bispectrum reflects non-linear interactions between signals at three different frequencies.

The apparent difficulty to interpret bicoherence has many researchers prefer more intuitive estimates of phase-amplitude coupling (PAC), like the one suggested by Canolty et al. (2006), despite PAC being the exact type of coupling measured by bicoherence, as well (Hyafil, 2015; Kovach et al., 2018). Estimates of PAC have even been shown to represent a special case of bicoherence, which is limited to interactions between a maximum of two as opposed to three sources (Shahbazi-Avarvand et al., 2018). While the bispectrum occurs naturally as the Fourier-transformed third-order cumulant-generating function, the mathematical formulation of the PAC estimator in Canolty et al. (2006) was driven by the need to quantify a particular type of coupling. The resulting estimator relies on bandpass filtering of the signal and makes two irreconcilable bandwidth demands: While the amplitude-giving signal needs to be filtered widely enough to preserve potential modulation side bands, the extraction of instantaneous phases and amplitudes requires narrow filtering. In comparison, bicoherence estimates do not rely on repeated bandwidth-filtering, they avoid the bandwidth-tradeoff allowing them to provide PAC estimates at a higher frequency-resolution and lower computational cost (Kovach et al., 2018; Shahbazi-Avarvand et al., 2018).

In addition, CFC measures based on higher-order spectra can be tweaked to selectively react to true signal interactions, while neglecting artifacts of volume conduction. A CFC measure of this kind was proposed by Chella et al. (2014) and was constructed by performing an antisymmetrization operation on two of the electrode indices in *B*_*ijk*_. Here, we extend this to a trivariate version, which not only vanishes for entirely independent source signals, but for interactions between less than three sources, as well. We thus construct a CFC measure, which is selective to the number of interacting signals.

In Schneidman et al. (2006) and Tkacik et al. (2014) it is shown that simple pairwise interaction models, in the latter case with an additional global constraint, can explain most of the coupling between small groups of neurons in the retina. If this was true for larger networks as well, the restriction to pairwise interactions could avoid the “curse of dimensionality” when using coupling models of ever-increasing statistical order. Unfortunately, this does not seem to be the case (Ganmor et al., 2011). Our approach is complementary to that and focusses on isolating those parts of observed interactions, which are strictly inconsistent with pairwise couplings. We believe this to be a more practical approach to the study of complex interactions.

Much of this paper is devoted to the statistical properties of this newly introduced coupling measure, which we derive from the statistical properties of complex variables using the central limit theorem. Our findings are of general importance for the statistical analysis of complex valued coupling measures. In a last step, we apply the coupling measure to EEG resting state data for healthy controls and patients with schizophrenia.

## 2. Methods

### 2.1. Background: Cross-Bispectra and Bicoherence

A cross-bispectrum is a tensor defined for three different signals, which represents coupling between signal components at three different frequencies. Let *X*_*i*_(*f*) be the Fourier coefficient of a signal in channel *i* at frequency *f* in some segment of data. Omitting the segment index, the cross-bispectrum is defined as

(1) $$\begin{array}{r} B_{ijk}\left(f_{1},f_{2}\right)=\langle X_{i}\left(f_{1}\right)X_{j}\left(f_{2}\right)X_{k}^{*}\left(f_{1}+f_{2}\right)\rangle , \end{array}$$

where 〈·〉 is the expectation which we approximate by an average over segments. The cross-bispectrum is a third-order statistical moment in the Fourier domain, analogous to the cross-spectrum, which is a second-order statistical moment. The frequency of the third signal in Equation (1) is constrained to be the sum of the first two frequencies, since any other choice leads to vanishing results in case of resting-state data, which, in contrast to task-related data, does not have an intrinsic clock.

Bicoherence is a normalized version of cross-bispectra. We here use a normalization based on three-norms rather than conventional two-norms (Shahbazi-Avarvand et al., 2014). This norm is defined as

(2) $$\begin{array}{r} N_{i}\left(f\right)={\langle |X_{i}\left(f\right){|}^{3}\rangle }^{1/3}, \end{array}$$

so that bicoherence is defined as

(3) $$\begin{array}{r} b_{ijk}\left(f_{1},f_{2}\right)=\frac{B_{ijk}\left(f_{1},f_{2}\right)}{N_{i}\left(f_{1}\right)N_{j}\left(f_{2}\right)N_{k}\left(f_{1}+f_{2}\right)}. \end{array}$$

Using this norm, the absolute value of bicoherence is bounded by one and the norm is constructed as a product of univariate norms, while other commonly used norms violate either of these two conditions.

### 2.2. Totally Antisymmetric Cross-Bispectrum (TACB)

It was shown by Chella et al. (2014) that a specific antisymmetric combination of the cross-bispectrum, namely

(4) $$\begin{array}{r} {\tilde{B}}_{ij}\left(f_{1},f_{2}\right)=B_{iij}\left(f_{1},f_{2}\right)-B_{iji}\left(f_{1},f_{2}\right), \end{array}$$

is robust to artifacts of volume conduction: if the signals in channel space are superpositions of independent sources, this quantity statistically vanishes. This is analogous to the case of the imaginary part of complex coherency, which is, apart from the imaginary unit, identical to its antisymmetric part (Nolte et al., 2004). In contrast to the imaginary part of coherency, ${\tilde{B}}_{ij}$ is not always purely imaginary but can have an arbitrary phase.

We take this idea one step further: Rather than including antisymmetry only with respect to two indices, we construct a totally antisymmetric part of the cross-bispectrum

(5) $$\begin{array}{r} T_{ijk}=B_{ijk}+B_{kij}+B_{jki}-B_{jik}-B_{kji}-B_{ikj}, \end{array}$$

where we omitted the frequency variable *f*. We refer to this quantity as the 'Totally Antisymmetric Cross-Bispectrum' (TACB). *T*_*ijk*_ is antisymmetric with respect to switching any pair of channel indices *ijk*. Its important property is that it vanishes not only if all sources are independent, but also if interactions are pairwise. This implies, that a non-vanishing result necessarily reflects an interaction of at least three sources.

To show this, we first assume that there are only two sources and the signals in sensor space can be written as

(6) $$\begin{array}{r} X_{i}\left(f\right)=\overset{2}{\underset{p=1}{\sum }}a_{ip}s_{p}\left(f\right), \end{array}$$

with mixing coefficients *a*_*ip*_ and source activity *s*_*p*_(*f*). Then, the cross-bispectrum reads

(7) $$\begin{array}{r} B_{ijk}\left(f_{1},f_{2}\right)=\underset{pqr}{\sum }a_{ip}a_{jq}a_{kr}B_{pqr}^{S}\left(f_{1},f_{2}\right), \end{array}$$

where

(8) $$\begin{array}{r} B_{pqr}^{S}\left(f_{1},f_{2}\right)=\langle s_{p}\left(f_{1}\right)s_{q}\left(f_{2}\right)s_{r}^{*}\left(f_{1}+f_{2}\right)\rangle \end{array}$$

denotes the cross-bispectrum of the sources. The crucial point is that the source indices can only assume two different values for two sources. Hence, for any term in the sum of Equation (7) two of the source indices must be equal and the term cannot have an antisymmetric part. For example if *q* = *p*, the corresponding contribution reads $a_{ip}a_{jp}a_{kr}B_{ppr}^{S}\left(f_{1},f_{2}\right)$ which is symmetric with respect to switching the indices *i* and *j* and its antisymmetric part vanishes. Furthermore, the antisymmetric combination is a linear operation, and if the antisymmetric part of all terms in Equation (7) vanishes, then also the antisymmetric part of the sum vanishes.

Next we consider the case with more than two sources, which only interact in a pairwise manner. For this we need to assume that all activities have zero mean. First, we emphasize that third-order statistical moments are additive for independent activities because terms involving two or more independent activities must contain at least one of them linearly, e.g., for two independent signals *x*(*f*) and *y*(*f*) one has

(9) $$\begin{array}{r} \langle x\left(f_{1}\right)x\left(f_{2}\right)y^{*}\left(f_{1}+f_{2}\right)\rangle =\langle x\left(f_{1}\right)x\left(f_{2}\right)\rangle \langle y^{*}\left(f_{1}+f_{2}\right)\rangle =0, \end{array}$$

since $\langle y^{*}\left(f_{1}+f_{2}\right)\rangle =0$ for signals with vanishing mean. Note, that this additivity is lost beyond the third order, because two independent signals can both occur non-linearly.

Putting things together, for only pairwise interactions the cross-bispectrum is the sum of the cross-bispectra of each pair, which all have a vanishing TACB, such the TACB of their sum vanishes, as well. An important aspect is that for *f*_1_ = *f*_2_ the cross-bispectrum is symmetric with respect to switching the first two indices. Thus, to observe a totally antisymmetric part, we need to study the interaction of signals at three different frequencies *f*_1_, *f*_2_, and *f*_1_ + *f*_2_.

It is important to consider, that the normalization term to calculate bicoherence from cross-bispectra is not symmetric with respect to switching indices. Hence, the totally antisymmetric part of bicoherence does not necessarily reflect an interaction of at least three sources. To normalize TACB we use a statistical normalization described below.

The basis of the above finding is the quasistatic approximation. Within this approximation the mapping of sources to sensors is frequency independent. Our essential argument still holds for frequency dependent forward mapping provided that corresponding frequency dependent factors do not depend on space. If also this weaker condition is violated by a non-negligible amount the claim is in general not valid. We leave it here as a open question to what extent this is the case for EEG or MEG.

### 2.3. Correct Statistics for TACB and Other Phase-Based Coupling Measures

#### 2.3.1. The Distribution of the Coupling Measure Under the Null-Hypothesis

Estimators of coupling strength fluctuate around the true coupling value (for unbiased estimators) and this also holds if there is no coupling between the signals at all. We therefore need to determine the distribution of coupling estimates for uncoupled signals to know whether our observed coupling value is evidence for coupling after all. A common approach is to estimate the null distribution using the surrogate data, which by definition should not be phase-coupled at all.

In general there are two approaches to the estimation of any distribution: Either we make prior assumptions on the type of distribution and only use the surrogates to estimate the distributions parameters, or we avoid any prior assumptions if we choose non-parametric tests. Non-parametric distribution estimates require a lot more data, but are often the only choice if prior distributional assumptions cannot be made. It is important to get a precise estimate of the null distribution of the coupling measure and it is even more important if reliable *p*-values are to be reported.

In the case of estimators which are defined as averages over complex quantities (e.g.), valid distributional assumptions can in fact be made by means of the central limit theorem: typical unnormalized coupling estimators, like the cross-bispectrum discussed here but also like a standard cross-spectrum, are defined as averages over complex values and are subject to the complex central limit theorem, which states, that values taken by the estimator will follow a complex normal distribution. If the null hypothesis is true and if the phases themselves are uniformly distributed, the complex estimator, say *u*, is distributed with the density

(10) $$\begin{array}{r} p_{u}\left(u\right)\sim \exp\left(\frac{-|u{|}^{2}}{2\sigma^{2}}\right) \end{array}$$

where σ is the only parameter and can be estimated empirically with surrogate data for which the null hypothesis is true using

(11) $$\begin{array}{r} \sigma^{2}=\frac{1}{2}\langle |u{|}^{2}\rangle \end{array}$$

It is well-known that in this case the absolute value of *u* is Rayleigh distributed

(12) $$\begin{array}{r} p_{\left|u\right|}\left(\left|u\right|\right)=\frac{\left|u\right|}{\sigma^{2}}\exp\left(\frac{-|u{|}^{2}}{2\sigma^{2}}\right) \end{array}$$

If we observe a specific coupling *u*_0_ we can hence estimate a *p*-value, i.e., the probability that this or a larger coupling was observed by chance if the null-hypothesis is true,

(13) $$\begin{array}{r} p=\int_{x=|u_{0}|}^{\infty }p_{\left|u\right|}\left(x\right)dx=\exp\left(-\frac{|u_{0}{|}^{2}}{2\sigma^{2}}\right) \end{array}$$

#### 2.3.2. Surrogate Data

While our TACB estimator *T* is known to generate Rayleigh distributed coupling estimates, we nonetheless generate some surrogate times series ${\tilde{X}}_{1}\left(f\right),\ldots {\tilde{X}}_{N}\left(f\right)$, following the approach of Canolty et al. (2006) to estimate the distribution's shape parameter σ: Given that TACB detects non-random phase relations between the frequency components *X*_*i*_(*f*_1_),*X*_*j*_(*f*_2_) and *X*_*k*_(*f*_3_), we destroy a potential phase structure by circularly shifting the *N*_*e*_ epochs of the third frequency component (_*X*_3_)1…*N*_*e*__ by a uniformly distributed offset within [1 … *N*_*e*_ − 1]. We used the TACB values ${\tilde{t}}_{ijkm}$ for the *m*.*th* surrogate data set for the channel triple *i, j, k*. We used *M* = 100 surrogate data sets to compute an estimate of the squared Rayleigh parameter σ^2^, for which an unbiased maximum likelihood estimate is given as

(14) $$\begin{array}{r} \sigma_{ijk}^{2}=\frac{1}{2M}\overset{M}{\underset{i=1}{\sum }}|{\tilde{t}}_{ijkm}{|}^{2} \end{array}$$

and defined a squared scaled TACB as,

(15) $$\begin{array}{r} Q_{ijk}=\frac{|t_{ijk}{|}^{2}}{2\sigma_{ijk}^{2}} \end{array}$$

#### 2.3.3. Comparison With z-Scoring

**Figure 1:**
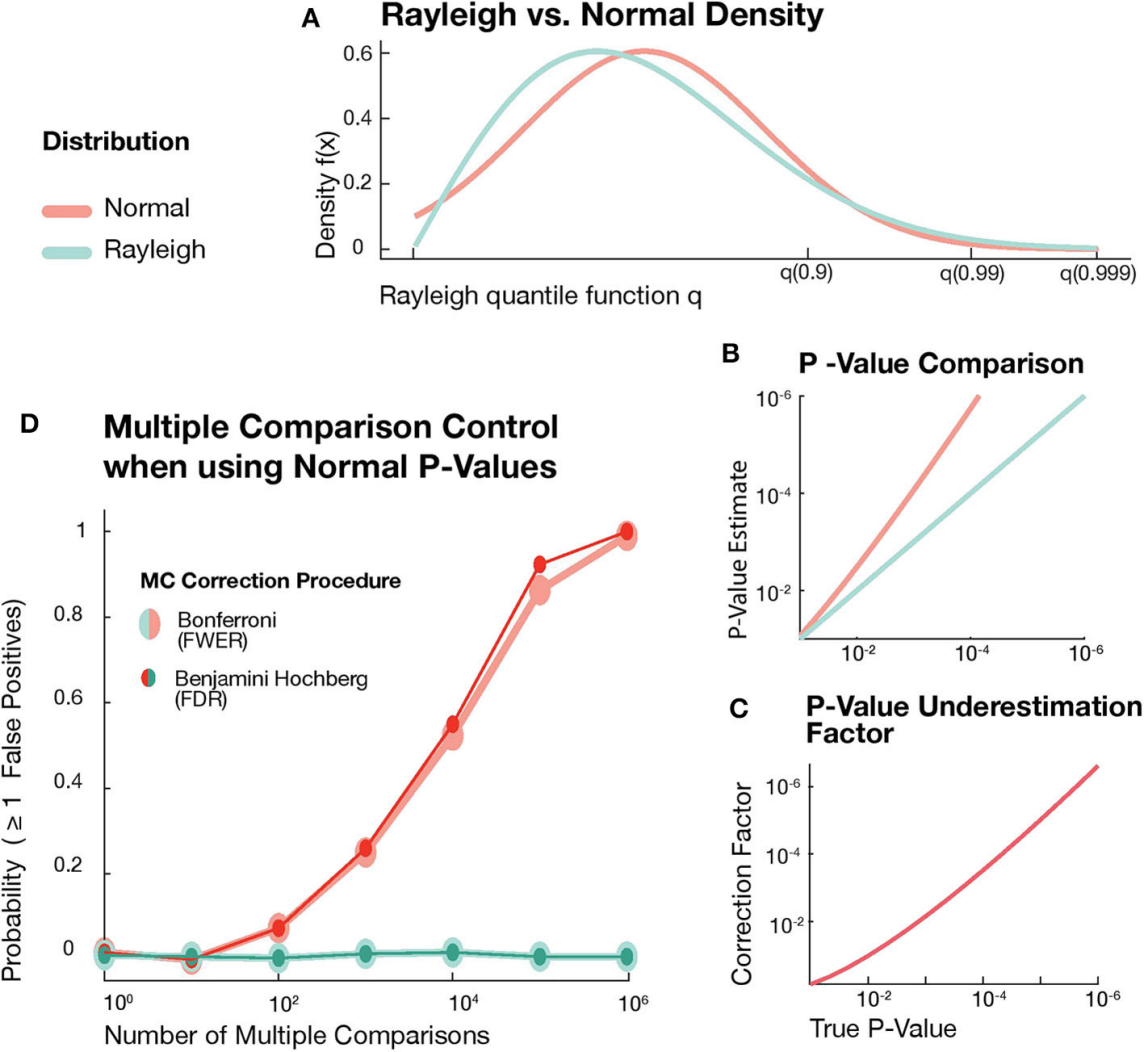
**(A)** Rayleigh and Normal distribution densities over Rayleigh quantiles. **(B)** Comparison between correct *p*-values from Rayleigh distribution (green) and the *p*-values assuming a Gaussian distribution. **(C)** Probability of at least one false detection after correction for multiple comparison as function of the number of comparisons. **(D)** Error accumulation of *p*-value estimation errors in the case of multiple comparisons for Bonferroni FWER control (big markers) and Benjamini-Hochberg FDR control (small markers).

A different statistical approach was proposed by Canolty et al. (2006): The empirical absolute value of the coupling is z-scored by subtracting mean and dividing by the standard deviation of the corresponding surrogates. We suppose that the authors calculated *p*-values, assuming, that under the null-hypothesis the z-score follows a standard Gaussian distribution. The difference between a Rayleigh and a Normal distribution might seem minor at first. But the consequences of the resulting *p*-value estimation errors can be very problematic for reasons:

1. It is not uncommon for researchers to state and compare very precise and often tiny *p*-values. When *p*-values are based on a Normal instead of a Rayleigh distribution Figure 1A, the estimation error increases for decreasing *p*-values (Figures 1B,C).
2. This underestimation of *p*-values might be considered negligible if we conduct a single hypothesis test. For multiple simultaneously tested hypotheses estimation errors accumulate and render multiple testing correction procedures ineffective. These procedures control the risk of false positives by keeping the FWER or FDR below a predefined threshold. For correctly estimated *p*-values the risk of false positives can be reliably kept below this threshold. But even small individual *p*-value estimation errors cause these control procedures to fail for higher numbers of simultaneously tested hypotheses.

To demonstrate this effect, we used the MATLAB built-in random number generator to draw sets of independent Rayleigh samples, where each of these sets represented a family of coupling estimates under the null hypothesis (absence of coupling). For each family of estimates we calculated the correct Rayleigh-based *p*-values, but also estimated *p*-values resulting from the mistaken assumption of normally distributed coupling estimates. To compute these normal-based *p*-values for a set of Rayleigh samples, we calculated a z-score *z*_*x*_ for each sample x and used the complementary error function erfc to estimate $p=0.5\text{erfc}\left(z_{x}/\sqrt{2}\right)$. Both sets of *p*-values were then corrected for multiple comparisons by either controlling FWER (Bonferroni) or FDR (Benjamini-Hochberg step-up) at a level α = 0.05 (green lines in Figure 1D). FDR and FWER are equivalent under the global null hypothesis and in both cases—presuming correctly estimated *p*-values—we can expect to observe any false positives in only 5% of the tested families. For an estimate of this percentage for our two sets of *p*-values, we performed 500 independent repetitions of the above procedure for 7 different hypothesis family sizes log-spaced between 10^0^ and 10^6^. Using normal-based *p*-values, the probability of false positives quickly increases for higher numbers of multiple comparisons (red lines in Figure 1D). For a family size of 10^6^ hypotheses, the probability of getting one or more false positives after correcting for multiple comparisons still is higher than 0.99. Thus, despite the absence of any true effect, we almost certainly get one or more significant results due to the minor but systematic underestimation of *p*-values.

### 2.4. TACB Analysis of EEG Alpha Oscillations

#### 2.4.1. Experimental Setup and Data

To demonstrate a use case of TACB, we analyze the same data as in Andreou et al. (2015a,b) and Shahbazi-Avarvand et al. (2018). The EEG recordings were provided by the Department of Psychiatry of the University Medical Center Hamburg-Eppendorf. The data comprises continuous resting-state recordings (5–10 min, sampled at 1 kHz, eyes closed) of 22 patients with first-episode schizophrenia and 24 healthy controls. Patients were recruited through the Center for Psychotic Disorders of the Department of Psychiatry, while controls were taken from the general public according to predefined inclusion/exclusion criteria regarding their medical history (Leicht et al., 2015). The data was recorded using 64 Ag/AgCl electrodes positioned according to the 10–20 system with additional electrode positions AF7,AF3, AF4, AF8, F5, F1, F2, F6, F10, FT9, FT7, FC3, FC4, FT8, FT10, C5, C1,C2, C6, TP7, CPz, TP8, P5, P1, P2, P6, PO3, POz, and PO4 mounted on an EEG cap (ActiCaps, Brain Products, Munich, Germany), Impedance was kept below 5 kΩ throughout the experiments and EEG data was recorded using the Brain Vision Recorder software version 1.10 (Brain Products, Munich, Germany). Post-processing involved ICA decomposition, artifact-removal by visual inspection and application of a 0.1–70 Hz bandpass filter. Furthermore, the data was down-sampled to 256 Hz and re-referenced to the common average reference.

#### 2.4.2. Subject-Specific Alpha Frequencies

**Figure 2:**
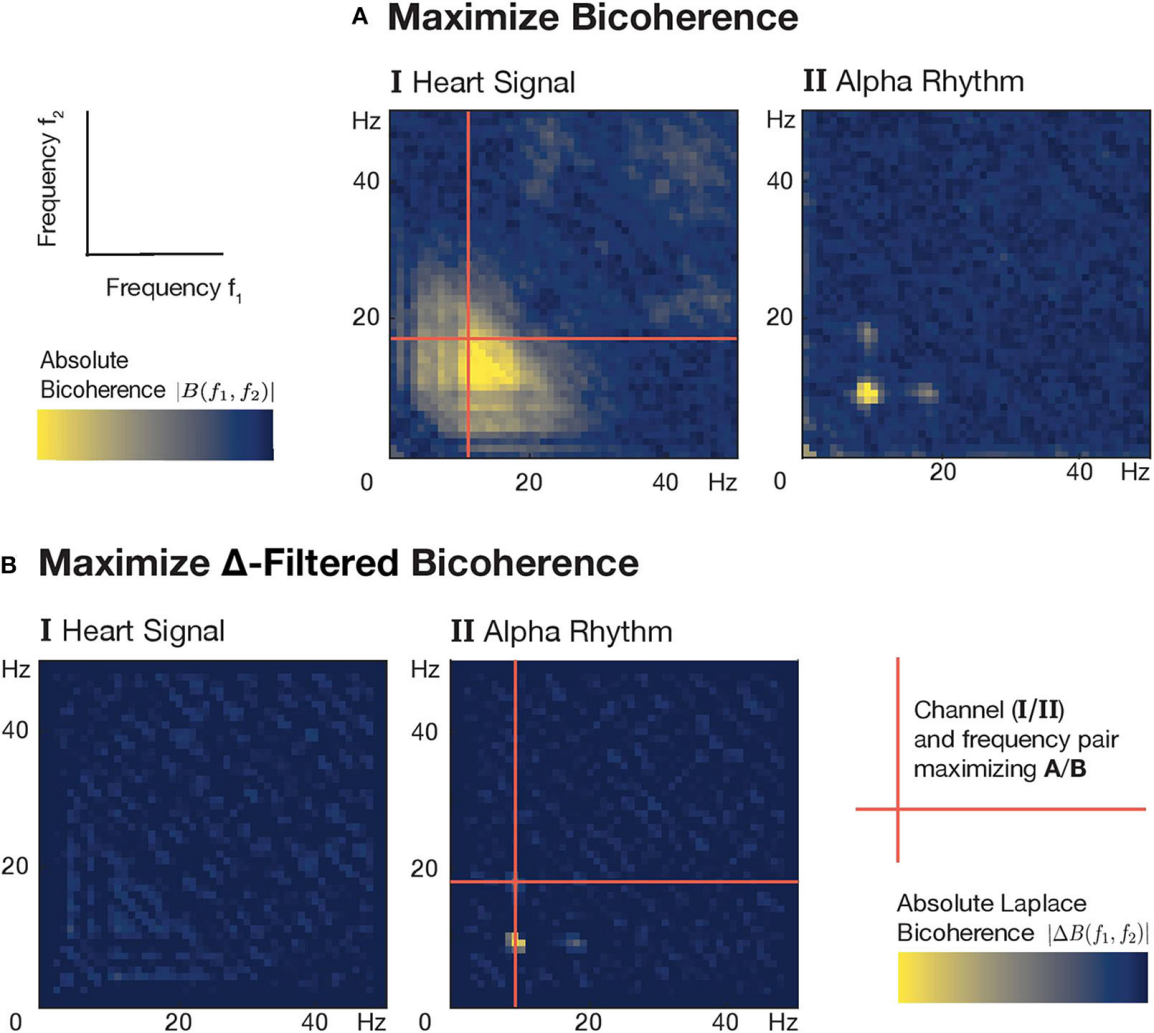
Algorithmic frequency and channel selection for alpha rhythm analysis in a subject with strong heart artifacts. **(A)** The presence of heart artifacts with high broadband bicoherence values (I) corrupts the channel and alpha frequency selection based on the maximization of bicoherence values. Despite the clear differences between the signals (I/II), the peak bicoherence value is achieved by the heart signal. **(B)** Application of a Laplace filter—also commonly used for edge detection in images—attenuates those changes in bicoherence values that gradually evolve over several neighboring frequency pairs (A/B.I). Relative to that it also enhances sudden peak-like changes in bicoherence values like they typically occur in patterns of alpha oscillations (A/B.II).

The fundamental frequency of alpha rhythms in humans is subject-specific and typically ranges from 9 to 13 Hz. To determine an individual alpha frequency for each of the 24 subjects, we devised an automated selection procedure, which makes use of the distinct visibility of alpha rhythm harmonics in univariate channel bicoherence patterns (Figure 2). These are auto-bicoherences—computed from a single channel signal and evaluated over several frequency pairs (*f*_1_, *f*_2_). While one could simply pick the channel and alpha frequency with the highest bicoherence peak value, this approach is hampered by the presence of heart artifacts, which themselves show large bicoherence values over several pairs of frequencies (Figure 2). The distinguishing feature between bicoherence patterns of alpha rhythms and those of heart artifacts is the occurrence of clearly localized peaks of the former. To quantify the peakiness of a point in the bicoherence plain—such a single point corresponds to the bicoherence calculated for a particular frequency pair at a single channel—it needs to be compared to neighboring points in that plain. The Laplace operator provides just that and is even used for edge detection purposes in image processing tasks. We therefore applied a discrete Laplace filter to the univariate channel bicoherences $b_{i}^{u}$ and selected the optimal channel *i* and alpha frequency *f*_α_ to maximize the new quantity $\Delta b_{i}^{u}$, thus

(16) $$\begin{array}{r} \left(i_{max},f_{max}\right)=\underset{i,f\in F}{\text{arg max}}\Delta b_{i}^{u}\left(f,2f\right), \end{array}$$

where *F* denotes the alpha-band band 9 and 13 Hz and where we approximated the discrete Laplace operator Δ for an offset of *h* = 2Hz approximated by finite differences as

(17) $$\begin{array}{r} \Delta b_{i}^{u}\left(f_{1},f_{2}\right)=\frac{1}{h^{2}}(b_{i}^{u}\left(f_{1}-h,f_{2}\right)+b_{i}^{u}\left(f_{1}+h,f_{2}\right)\\ \;+b_{i}^{u}\left(f_{1},f_{2}-h\right)+b_{i}^{u}\left(f_{1},f_{2}+h\right)-4b_{i}^{u}\left(f_{1},f_{2}\right)). \end{array}$$

Finally, to estimate TACB on real EEG data we proceeded according in the following steps: We estimated TACB values *T*_*ijk*_ for all antisymmetric triples with electrode indices *ijk*, which amounted to *N*_*e*_ = 35990 unique triples without repeating electrode indices. For the original pre-processing the data were divided into epochs of 2 s duration and epochs containing strong outliers were removed. Correspondingly, for a specific electrode triple we divided the channel recordings into epochs of 512 samples (i.e., 2 s duration), each of which we further divided into five overlapping segments of 256 samples with 75% overlap. The reason for the division of the data into both epochs and segments was to avoid using segments which overlap across different epochs when intermediate epochs were taken out in the pre-processing. To each segment ${\left(x(t)\right)}_{\text{seg}}$ we applied a Hann window and computed its Fourier transforms ${\left(X(f)\right)}_{\text{seg}}$. The cross-bispectrum at a frequency triple (*f*_1_, *f*_2_, *f*_3_) was then approximated by an average over all segments across all epochs. The totally antisymmetric part was calculated as defined in Equation (5).

## 3. Results

### 3.1. Simulations

To illustrate the difference between the full cross-bispectra and TACB we simulated EEG for 64 electrodes for three signals using the same EEG system as analyzed. The first signal, *x*_1_(*t*) was constructed as white noise filtered around 10 Hz with a filter width of 1 Hz. The second and third signal were constructed as $x_{2}\left(t\right)=x_{1}^{2}\left(t\right)$ and $x_{3}\left(t\right)=x_{1}^{3}\left(t\right)$. All three signals were normalized to 1 using the *L*_2_-norm. These signals were assigned to source activities in different ways as explained below. The sources were placed 4 cm below electrodes C3, C4, and Cz, pointing into radial direction, where the radial direction was defined to be the direction of the surface normal at the corresponding electrode location. The forward calculation was done for a three shell realistic head shape. The code is available in the “MEG and EEG Toolbox of Hamburg” which can be downloaded at https://www.uke.de/english/departments-institutes/institutes/neurophysiology-and-pathophysiology/research/research-groups/index.html.

The three signals *x*_*i*_(*t*) were assigned to source activities *s*_*i*_(*t*) depending on the choice for the number of active sources. For one active source we chose

(18) $$\begin{array}{r} s_{1}\left(t\right)=x_{1}\left(t\right)+x_{2}\left(t\right)+x_{3}\left(t\right)\\ s_{2}\left(t\right)=0\\ s_{3}\left(t\right)=0 \end{array}$$

For two active sources the choice was

(19) $$\begin{array}{r} s_{1}\left(t\right)=x_{1}\left(t\right)+x_{2}\left(t\right)\\ s_{2}\left(t\right)=x_{3}\left(t\right)\\ s_{3}\left(t\right)=0 \end{array}$$

and for three active sources the choice was

(20) $$\begin{array}{r} s_{1}\left(t\right)=x_{1}\left(t\right)\\ s_{2}\left(t\right)=x_{2}\left(t\right)\\ s_{3}\left(t\right)=x_{3}\left(t\right) \end{array}$$

**Figure 3:**
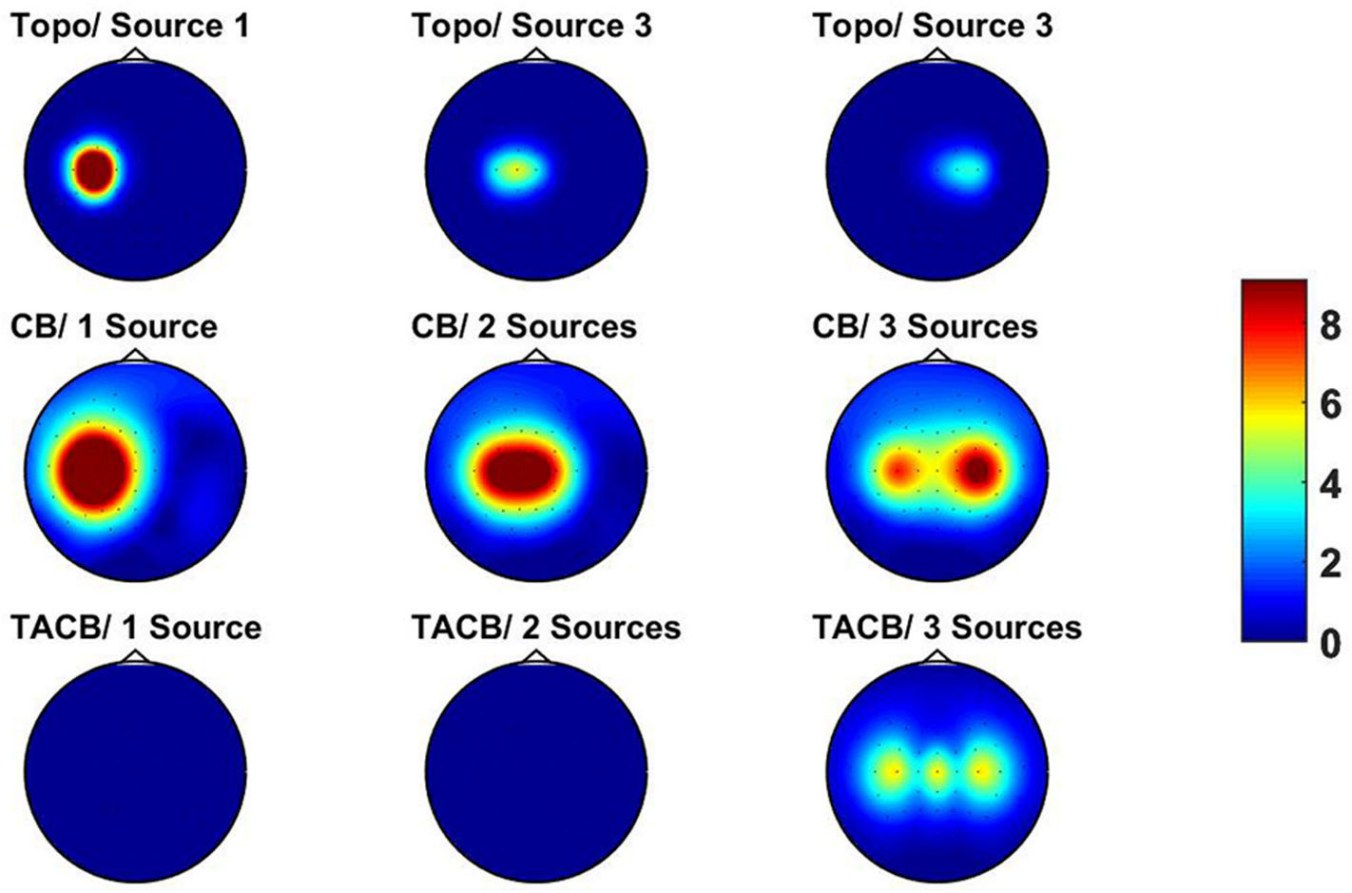
Results for simulated data. Top row: topographies for three sources. Middle row: absolute value of cross-bispectra (CB) maximized over two of the three channel indices. Left panel: all activities are assigned to one source. Middle panel: all activities are assigned to two sources. Right panel: the activities are assigned to three sources. Bottom row: same as middle row for TACB. For the left and middle panel the signal in sensor space vanishes up to Matlab rounding errors.

After mapping the source activities to sensor space we calculated cross-spectra and TACB at frequencies *f*_1_ = 10 Hz and *f*_2_ = 10 Hz. Results are shown in Figure 3. In the top row we show the three topographies. For the cross-bispectra and TACB we always maximize the absolute values across two out of the three sensor indices. In contrast to the cross-spectra we observe that TACB is only non-vanishing if three sources are active.

### 3.2. Empirical Results for the Totally Antisymmetric Part of Cross-Bispectra

After determining alpha-rhythm frequencies for each subject, we calculated *p*-values for the existence of a totally antisymmetric part of the cross-bispectrum. The selection of frequencies was based on univariate bicoherence, and all univariate quantities vanish identically for the totally antisymmetric part. Thus, by the chosen selection scheme we did not bias the results toward large antisymmetric parts. To correct for multiple comparison we used the false discovery rate at an alpha-value of 0.05, such that 95% of all detections can be expected to be true detections, calculated a corresponding threshold, and considered all *p*-values above that threshold as insignificant. In 10 out of 24 subjects we made significant detections after FDR correction, and the total number of significant detections was 30,438. Of course, these numbers slightly vary when reanalyzing the data, because the statistical test is based on random permutations. We also pooled all *p*-values and applied FDR correction to the pooled set. We found that 7,620 out of 5,447,544 channel triples survived correction for multiple comparisons. To visualize and average the results across subjects channels we use the scaled values as defined in Equation (15). Let *Q*_*i,j,k,m*_ be that value for channel triple *i, j, k* and subject *m*. Then we calculate a subject average for *M* subjects as

(21) $$\begin{array}{r} {\tilde{Q}}_{i,j,k}=\frac{1}{M}\underset{m}{\sum }Q_{i,j,k,m} \end{array}$$

**Figure 4:**
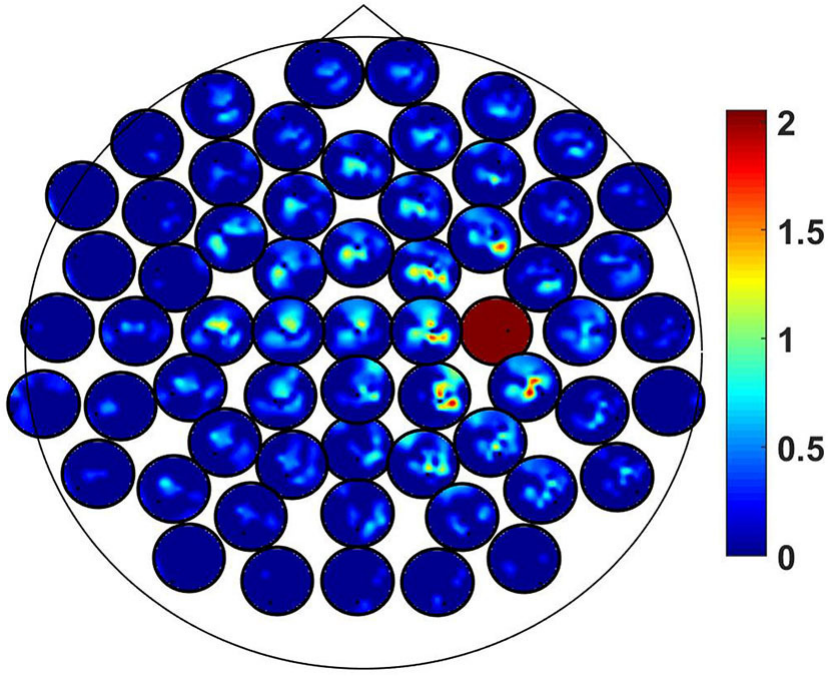
Topographic plot of the matrix ${\tilde{Q}}_{ijk}$ defined in Equation (21) for fixed last index. Each small circle contains one row of that matrix. The full red circle indicates the fixed channel.

This tensor is difficult to display graphically. To illustrate the result we fixed the k.th channel to *k*_0_ chosen as the one which maximizes $\tilde{Q}$ across all channel triples. For fixed channel *k* we can display the remaining matrix similar to coherence (Nolte et al., 2004). The result is shown in Figure 4 where the chosen channel *k*_0_ corresponds to the red circle which reflects the property of TACB that ${\tilde{Q}}_{i,k,k}=0$, i.e., it must vanish if two indices are equal. The maximizing channel was found to be over the right motor area and the interacting channels are in the vicinity of that.

### 3.3. Healthy Controls vs. Schizophrenic Patients

**Figure 5:**
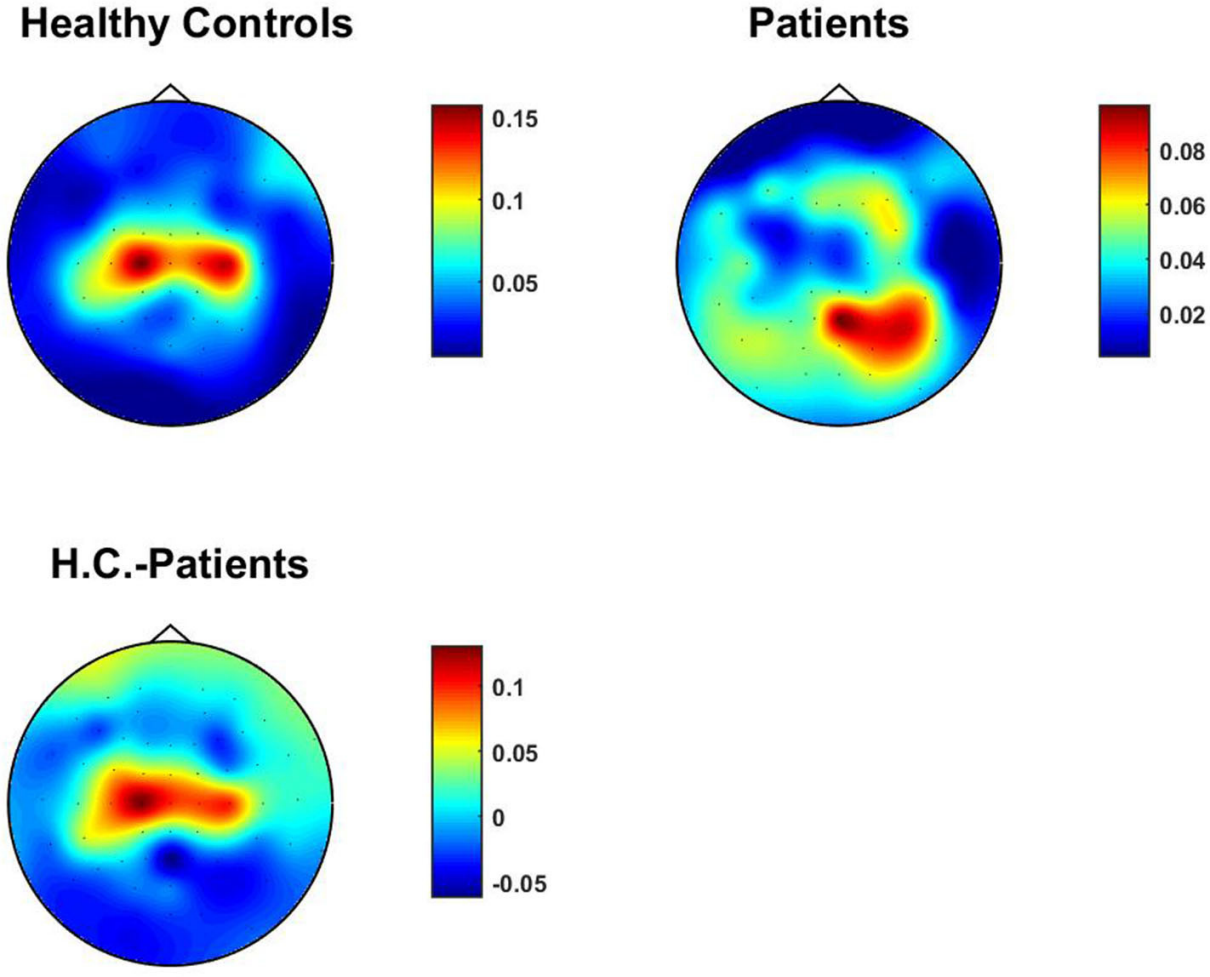
TACB averaged over subjects and for each channel over all other channels.

We studied TACB both for the 24 healthy subjects and 22 patients with schizophrenia. We found that in 13 out of 22 patients detections of coupling after FDR corrections. To illustrate results we not only average over subjects but also across two of the three channel indices.

(22) $$\begin{array}{r} {\hat{Q}}_{i}=\frac{1}{MN^{2}}\underset{m,j,k}{\sum }Q_{i,j,k,m} \end{array}$$

for *N* channels and *M* subjects. Results for this topographical map for healthy controls, patients with schizophrenia and the difference are shown in Figure 5. For healthy controls we observe the strongest signals over left and right motor areas. For patients this effect is attenuated such that signals coming from occipital areas are more apparent. This result is analogous to results for univariate bicoherence presented by Shahbazi-Avarvand et al. (2018), where, in contrast to the present analysis, univariate bicoherence was calculated at *f*_1_ = *f*_2_ set in the alpha range. We recall that such a frequency choice is meaningless for TACB analysis as it vanishes if two of three frequencies are equal. We tested whether the means are significantly using a permutation test using *M* = 10, 000 permutations, and corrected the *p*-values for all sensors using the false discovery rate. While results are qualitatively similar, we did not find that the difference is significant, even though the data set was identical, which could be due to the fact that couplings between three frequencies is less robust than plain alpha-beta coupling.

**Figure 6:**
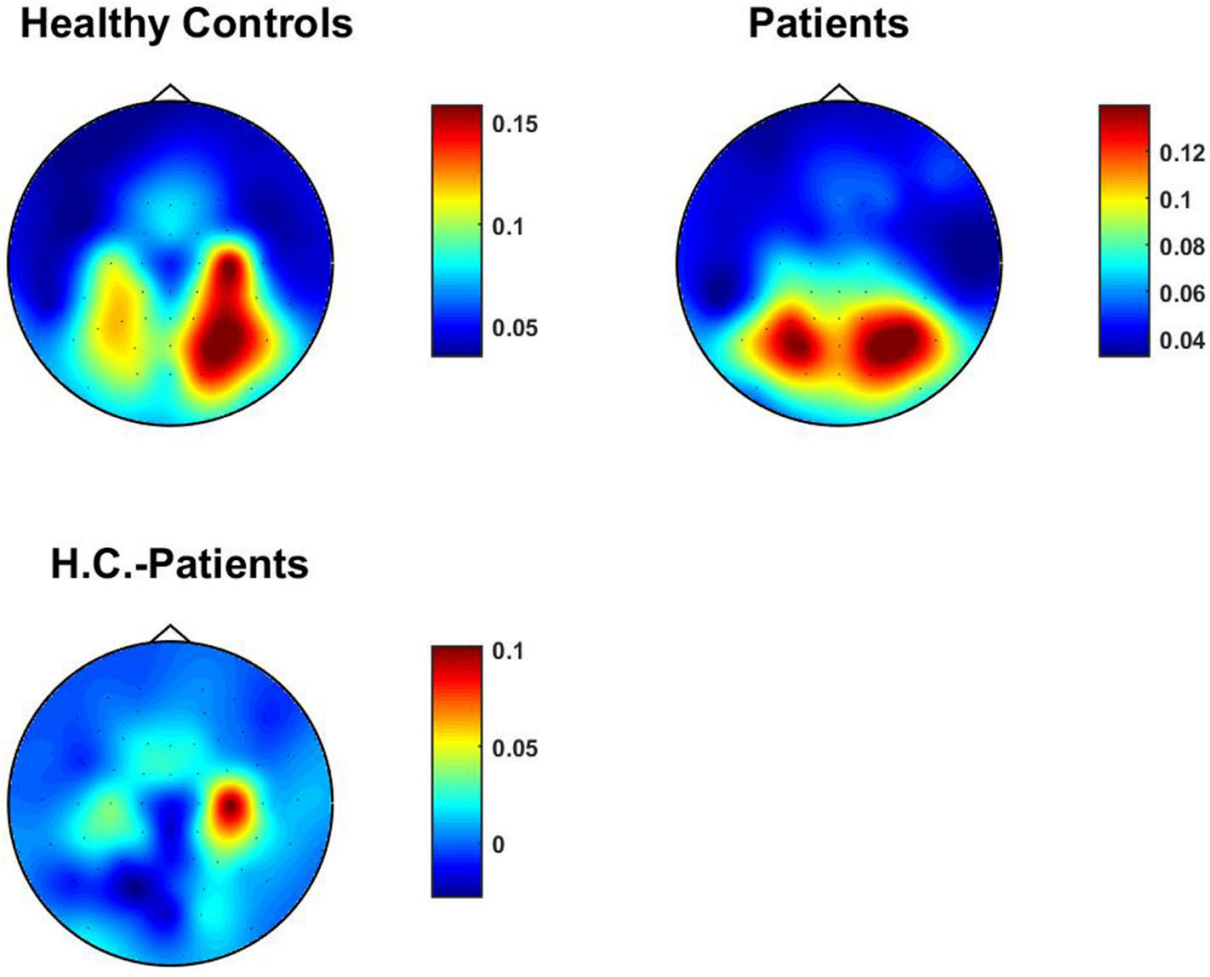
Absolute value of univariate bicoherence at selected frequency combinations averaged over subjects.

We also calculated univariate bicoherence at the selected frequencies for all channels and averaged them over subjects. In contrast to TACB, the bicoherence is normalized already and it is not necessary to use a statistical normalization. Results are shown in Figure 6 which is qualitatively similar to TACB. However, these univariate quantities do not necessarily reflect interactions between different sources. Also, for the univariate coupling estimates we did not find the difference to be significant.

## 4. Conclusion

We derived a new measure of brain connectivity consisting of the totally antisymmetric part of cross-bispectra, the general third order statistical moments in the frequency domain. It has the property that it vanishes apart from statistical fluctuations for any mixture of independent and pairwise interactions. It is hence a measure of interaction between at least three sources. This property distinguishes it from all other known measures of brain connectivity robust to artifacts of volume conduction which are bivariate measures and in general do not vanish for pairwise interactions. This may give a window to study more complex phenomena of brain interactions without risking that estimated multivariate properties of brain interaction are again an artifact of volume conduction.

This is mainly a conceptual paper presenting the method itself and showing that we can observe significant non-vanishing coupling on channel level. To be applicable it requires very special features of the data, namely the existence of coupling between three different frequencies as observable within EEG data for the alpha rhythm with two higher harmonics. To our knowledge for resting state data, the alpha rhythm is the only candidate for that, and also there the second higher harmonic is typically much weaker than the first higher harmonic.

For the statistical analysis we developed a, to our knowledge, new approach, exploiting the fact that cross-bispectra are averages across a large number of segments and hence approximately Gaussian distributed in the complex domain. As a consequence its absolute value is approximately Rayleigh distributed under the null-hypothesis and it has only one free parameter which can be estimated from surrogate data. This substantially deviates from alternative approaches where the absolute-value is z-scored. We showed in simulations that the latter approach can heavily underestimate *p*-values and can lead to too many false positives. We emphasize that this approach is applicable to all cases of a complex coupling measures which are calculated as an average over a large number of trials. In particular, it is applicable also to the cross-spectrum but not to coherence since the latter is constructed from averages (cross-spectra and power) but is not an average itself. While this seems to limit the applicability of this approach we note that, e.g., coherence is different from zero if and only if the cross-spectrum, the numerator of coherence, is different from zero. It is hence sufficient to study the numerator only having a simple Gaussian distribution in the complex domain.

We applied the methods to EEG data of healthy controls and patients with schizophrenia and found significant coupling mostly for healthy subjects for left and right motor areas, but we could not find significant differences, which we believe is due to the fact that this kind of coupling is rather weak. While we cannot claim that this coupling is useful as a biomarker it is conceivable that it improves diagnostics when combined with other biomarkers. An open question, which will be addressed in the future, is the estimation of coupling in the source space. It is necessary to develop special techniques for this beyond the scope of this paper, as a straight forward approach to estimate coupling between all triples of voxels is computationally far too costly.

## Data Availability Statement

The data analyzed in this study is subject to the following licenses/restrictions: for the availability of the datasets generated and analyzed during the current study Christina Andreou needs to be contacted. Requests to access these datasets should be directed to christina.andreou@uksh.de.

## Ethics Statement

The studies involving human participants were reviewed and approved by Ethics committee of the Hamburg Chamber of Physicians. The patients/participants provided their written informed consent to participate in this study.

## Author Contributions

SB, CA, and GN have analyzed the data. The theory was developed by SB and GN. The paper was written by SB and GN, and revised by CA. All authors contributed to the article and approved the submitted version.

## Conflict of Interest

The authors declare that the research was conducted in the absence of any commercial or financial relationships that could be construed as a potential conflict of interest.
